# Evolution and Effects of Caffeine Utilization Throughout Medical and Surgical Training

**DOI:** 10.7759/cureus.77600

**Published:** 2025-01-17

**Authors:** Celeste Shoeleh, Reagan Sandstrom, Daniel P Pierce, Trushar Patel

**Affiliations:** 1 Department of Urology, University of South Florida Morsani College of Medicine, Tampa, USA

**Keywords:** caffeine consumption, medical student health, occupational stress, perceived wellness, resident well-being, stimulant use

## Abstract

Objective

The objective of the study is to investigate the evolution of caffeine consumption during medical education, its variation by specialty, and any potential negative impacts on medical trainees.

Methods

We employed a multi-institutional, anonymous questionnaire to gather data on caffeine consumption and related effects among medical trainees. The survey quantified caffeine intake and its impact on sleep, focus, and fatigue. Comparisons were drawn between medical students and residents. Statistical analysis was conducted using chi-squared tests and T-tests.

Results

Totaling 315 survey respondents, 211 (67%) were medical students and 104 (33%) were residents. Among resident respondents, 44 (42.3%) were surgical residents and 60 (57.7%) were medicine residents. Residents demonstrated more caffeine consumption compared to medical students (200 mg vs 152 mg, p = 0.001); no significant difference in consumption was found between surgical and medicine residents (176 mg vs 217 mg, p = 0.112). An inverse correlation was identified between caffeine intake and self-reported daily tiredness for student/resident groups (p = 0.024 and p = 0.023, respectively). No significant associations were found between the year of medical training and tiredness (p = 0.503), focusing (p = 0.105), or sleep (p = 0.178).

Conclusion

We demonstrated that the year of medical training does not have an impact on caffeine usage or effects, and there is no significant difference between surgical/medical residents. There is a difference between residents and medical students with a significant impact on career-related parameters such as focus and sleep, underscoring the need to further investigate caffeine usage implications in medical trainees and its long-term effects on fatigue management.

## Introduction

Caffeine is a naturally occurring psychostimulant consumed globally in various forms for its stimulant properties and mood-enhancing effects, as well as part of cultural practices. Due to its mass availability in coffees, teas, and energy drinks, caffeine consumption is a mainstay in the medical profession. Many medical students and residents cope with the demanding lifestyle and long work hours with caffeine, but research on their patterns and consequences of use is limited. Current studies have cited several reasons for caffeine consumption in students and residents, including relieving stress, staying awake, driving, and exercising [1,2]. Significant consequences of caffeine use have also been studied and included dependency, withdrawal symptoms, poor sleep quality, anxiety, gastritis, aggression, and palpitations [1,3]. Concerning caffeine consumption in medical training, there is a lack of data exploring how caffeine consumption can change in medical training or vary based on medical subspecialty. Current literature has focused on individual medical training groups. A study examining a group of orthopedic residents found that caffeine consumption did not correlate with post-graduate year (PGY) but significantly differed between rotations like trauma or pediatrics [3].

This study explored caffeine use in various medical subpopulations across their training levels, including medical students, medicine residents, and surgical residents. Our objective was to trace consumption patterns and assess caffeine's effects on sleep duration, quality, fatigue, and concentration.

## Materials and methods

Survey

A 12-question survey (Appendices) was developed addressing medical trainees’ demographic information including sex, age, education year, and caffeine consumption with subjective daily tiredness, focus, and sleep quality. The questions were derived from common side effects of caffeine consumption; validated scales were not utilized in order to preserve survey length. The survey provided respondents with standardized information on caffeine content in commonly ingested caffeine sources including coffee, energy drinks, and pre-workout. The completed survey was sent via email, and responses were submitted anonymously through a Qualtrics-powered online survey (Qualtrics International Inc., Seattle, WA, US). Results were then categorized and analyzed based on several factors, such as the amount of caffeine consumed, and its impact on sleep, focus, and fatigue. These categories were further examined by year in medical education and by program type, including medical students, surgery/surgical subspecialty residents, and medicine/specialist residents.

Statistics

The mean daily consumption of caffeine in milligrams (mg) for medical students and residents was calculated by taking the mean value of each caffeine consumption category listed on the survey (e.g., respondents of 100-199 mg of caffeine category were given the value of 150 mg for mean consumption calculation). The resident group was subdivided into surgical and medical residents. Using the paired T-test for variation, the mean consumption of medical students and residents was directly compared, and the mean consumption of surgical residents and medical residents was compared. Daily caffeine consumption across all years of training (medical student 1 (MS1) through PGY5) was also analyzed. Data on "feeling tired," "difficulties focusing," and "nightly sleep quality" were categorized by group (medical student and resident) and analyzed against caffeine consumption using chi-squared tests. This analysis also compared years of medical education with subjective responses via chi-squared tests. The statistical analysis utilized data from 315 valid responses; however, the response rate could not be determined due to the survey's distribution method by respondents and institutions.

## Results

Study population

Out of the 315 survey respondents, 211 (67%) were medical students and 104 (33%) were residents. For medical students, 54 (17.14%) were MS1s, 60 (19.05%) were MS2s, 61 (19.37%) were MS3s, and 36 (11.43%) were MS4s. For residents, 41 (13.02%) were PGY1s, 26 (8.25%) were PGY2s, 18 (5.71%) were PGY3s, 13 (4.13%) were PGY4s, and six (1.90%) were >PGY5s. Among resident respondents, 44 (42.3%) were surgical residents and 60 (57.7%) were medicine/specialist residents. Of all respondents, 133 (42.2%) were men, 181 (57.5%) were women, and one (0.3%) was non-binary/third gender.

Caffeine consumption

The types of caffeine consumed by the trainees included coffee, tea, energy drinks, soda, pre-workout drinks, and other non-specified supplements. As expected, coffee, tea, and energy drinks were the most popular. These beverages typically contain 80-200 mg of caffeine per serving, with tea being the outlier at 26 mg per 8 oz cup. The types of caffeine consumed were similar between students and residents, and consumption was mostly in the morning.

The mean caffeine consumption for medical students was 152 mg (95% CI (136, 167)) and for all residents 200 mg (95% CI (176, 225)), and further divided, the mean caffeine consumption for surgical and medical residents was 176 mg (95% CI (142, 210)) and 217 mg (95% CI (183, 251)), respectively. Residents demonstrated significantly more caffeine consumption when compared to medical students (p = 0.001), while there was no significant difference in caffeine consumption when comparing surgical residents to medical/specialist residents (p = 0.112) (Table 1). There was no correlation between years of education and the amount of caffeine consumed (p = 0.089) (Table 2).

**Table 1:** Caffeine Consumption for Medical Students, Total Residents, Surgical Residents, and Medicine Residents Bold values are statistically significant. MS: medical students; Res: residents; Surg Res: surgical residents; Med Res: medicine residents

	MS mean	Res mean	Surgical Res mean	Medicine Res mean	p-value	CI (95%)
Caffeine consumption	152 mg	200 mg	-	-	0.001	MS (136, 167), Res (176, 225)
Caffeine consumption	-	-	176 mg	217 mg	0.112	Surg Res (142, 210), Med Res (183, 251)

**Table 2:** Caffeine Consumption by Amount Consumed Data in each column is the number of students self-reporting daily caffeine consumption from the given categories. MS: medical students; PGY: post-graduate year

Caffeine/day	None	<100 mg	100-199 mg	200-299 mg	300-399 mg	>400 mg	p-value
MS1	10	15	13	10	3	2	0.088
MS2	10	9	26	12	2	1	
MS3	9	7	23	11	6	5	
MS4	1	11	16	2	2	4	
PGY1	5	3	11	11	6	5	
PGY2	1	3	7	7	3	5	
PGY3	4	2	6	6	1	2	
PGY4	2	1	4	4	1	1	
>PGY5	2	2	1	1	0	0	

Tiredness

In the survey, 48.8% of medical students and 32.9% of residents reported often or always feeling daily tiredness. Caffeine consumption was linked to daily tiredness in both groups (p = 0.024 for students, p = 0.023 for residents) (Table 3). However, no significant relationship was found between the year of medical training and daily tiredness (p = 0.503).

**Table 3:** Trainees Who Experience Tiredness Values in bold are statistically significant. MS: medical students; Res: residents

	MS		Res	
Daily caffeine consumption	Often/always experience tiredness	Sometimes/rarely/never experience tiredness	Often/always experience tiredness	Sometimes/rarely/never experience tiredness
None	7	23	4	10
<100 mg	20	22	1	9
100-199 mg	42	35	16	11
200-299 mg	17	18	10	19
300-399 mg	10	3	7	4
>400 mg	6	6	8	5
Total	102 (49%)	107 (51%)	46 (44%)	58 (56%)
p-value	0.024		0.023	

Difficulties focusing

In the focus section of the survey, 52.6% of medical students and 32.7% of residents reported difficulties focusing at least three to four times per week. A significant negative correlation was observed between caffeine consumption and focus for medical students (p = 0.019), but not for residents (p = 0.108) (Table 4). Additionally, no significant relationship existed between years of medical education and focus difficulties (p = 0.105).

**Table 4:** Trainees Who Experience Difficulty Focusing The value in bold is statistically significant. MS: medical students; Res: residents

	MS		Res	
Daily caffeine consumption	Difficulty focusing 3-7 days/week	Difficulty focusing <3 days/week	Difficulty focusing 3-7 days/week	Difficulty focusing <3 days/week
None	14	16	2	12
<100 mg	18	24	3	7
100-199 mg	35	42	9	18
200-299 mg	24	11	7	22
300-399 mg	11	2	7	4
>400 mg	8	4	6	7
Total	110 (53%)	99 (47%)	34 (33%)	70 (67%)
p-value	0.019		0.108	

Sleep quality

Regarding medical students responding to the sleep quality portion of the survey, 114 (54.5%) were considered affected (responding “very poor,” “poor,” or “adequate” nightly sleep quality), and 95 (45.5%) were considered not affected. The amount of caffeine consumption had no statistically significant correlation with reported sleep quality for medical students and residents (p = 0.326 and p = 0.108, respectively) (Table 3). There was no correlation between the year of medical training and reported sleep quality (p = 0.178) (Table 5).

**Table 5:** Effects on Tiredness, Focus, and Sleep Across Medical Training MS: medical students; PGY: post-graduate year

	MS1	MS2	MS3	MS4	PGY1	PGY2	PGY3	PGY4	>PGY5	p-value
Tiredness (often/always)	26 (48%)	24 (41%)	30 (49%)	22 (61%)	17 (47%)	16 (62%)	6 (33%)	5 (38%)	2 (33%)	0.503
Focus (difficulty focusing 3-7 days/week)	30 (57%)	31 (52%)	34 (56%)	15 (42%)	13 (32%)	13 (50%)	4 (22%)	3 (23%)	1 (17%)	0.105
Sleep (very poor/poor/adequate sleep)	38 (70%)	32 (54%)	31 (51%)	14 (39%)	27 (66%)	14 (53%)	10 (56%)	7 (54%)	3 (50%)	0.18

## Discussion

Statement of principal findings

The demands of medical training and the increased stress placed on learners are a broad topic that has influenced research into how medical students and residents cope with the rigorous lifestyle. This current study utilized a multi-institutional survey to understand how caffeine consumption plays into the daily life of those in medical training and analyze how caffeine consumption may change as learners progress through their education. To our knowledge, this study is the first to identify how caffeine consumption differs between medical students and residents and compares surgical versus medicine/specialist residents. This study found that residents drink more caffeine than medical students, without any difference in the mean between medical and surgical residents. Fatigue was found to be associated with caffeine consumption, but there was no correlation between caffeine consumption and sleep hours. Medical students were more likely to report difficulties in concentration associated with caffeine than residents.

Interpretation within the context of the wider literature

This study found that the average caffeine consumption among medical students was 152 mg per day, similar to that of most Americans who drink 165 mg of caffeine on average daily [4]. Residents’ caffeine consumption was significantly higher, with a total average of 200 mg daily. This is an expected difference, as prior research has documented a correlation between increased work hours and caffeine consumption [5]. Medical students work under 60 hours per week, but residents typically work more hours and may consume additional caffeine to fulfill responsibilities [6-8].

When comparing surgical versus medicine residents, the mean consumption was not statistically significant, with surgical residents consuming 176 mg and medicine residents consuming 217 mg daily. Similar caffeine consumption among residents is unexpected, given the anticipation that caffeine intake would align with work hours. Notably, surgical residents work an average of 84.3 hours weekly, while medicine residents work 69.2 hours, suggesting a workload disparity [5,8]. Differences in work environments may contribute to unexpected caffeine consumption trends among residents. Medicine residents have been shown to spend double the amount of time on electronic health records than their surgical colleagues, which translates to increased desk time and more access to caffeine [9]. Surgical residents also spend a significant portion of their shift within the operating room, reducing their access to beverages. Regardless of the residency specialty or student status, most learners still consume less than the maximum recommended amount of 400 mg/day and, thus, are not at an increased risk for the toxic effects of caffeine compared to the general public [10].

Despite the spike in caffeine consumption from medical school to residency, caffeine consumption did not significantly change based on education year; each year of medical students shared similar consumption patterns, and each year of residents shared similar consumption patterns.

Medical students and residents demonstrated increased feelings of fatigue as caffeine consumption increased. The correlation between fatigue and consumption may result from prolonged wakefulness demanded by the medical field. Interestingly, despite increased fatigue, no correlation was found between caffeine use and decreased sleep hours in medical students or residents. Reduced sleep, regardless of caffeine intake, may be attributed to the medical field’s rigorous nature, which requires prolonged wakefulness to study the vast amount of material while balancing clinical duties, research projects, and home life responsibilities. Regardless of sleep hours, fatigue in medical trainees is a significant occupational hazard. Previous literature has highlighted the importance of acknowledging fatigue in trainees and its effect on burnout, personal well-being, and self-reported medical errors [11,12].

Notably, only medical students relayed the significant impact of caffeine on difficulty concentrating. Residents’ ability to maintain focus despite similar caffeine consumption and fatigue trends to medical students might reveal improved work skills as their education progresses, or there may be a biased perception of their capabilities. Prior studies have identified how resident physicians view fatigue as a personal obstacle manageable with training rather than an occupational threat [13]. Residents denying difficulties with concentration may have impaired working abilities of which they are unaware. This potentially harmful viewpoint may lead to further burnout and significant work errors.

Strengths and limitations

This study’s strengths included its focus on all levels of medical education and its broad geographical representation. However, there was no succinct method of distributing the survey or identifying the capture rate, which could lead to sample bias and conclusions not truly reflective of all learners in the United States. The participation rate was also limited despite having more than 300 respondents. Fewer senior residents completed the survey compared to interns or second-year residents, which could lead to inaccurate conclusions about the association between caffeine and level of training. Notably, residents who have been in training longer have been shown to have reduced work times and, thus, may not require as much caffeine as interns [14].

In creating an efficient survey, many demographic variables were not controlled outside of gender and age. Race, ethnicity, and smoking status were not identified in this study but may have contributed to differences in consumption. Prior trends have shown non-Hispanic White individuals and individuals who smoke to consume significantly more caffeine than their Hispanic or non-smoker counterparts [5]. Not accounting for these confounding variables could lead to correlations that may not exist.

It is also worth mentioning the limitations due to social desirability bias when addressing caffeine consumption. Socially, it may be undesirable to expose extremely high caffeine use in daily life, causing respondents to downplay their true caffeine intake. Social desirability bias may also play a role in how medical trainees respond to their ongoing difficulties focusing or tiredness in fear of seeming to be a poor or dangerous medical trainee.

Implications for policy, practice, and research

This survey has identified notable differences and similarities in caffeine consumption across multiple levels of medical training. However, due to limitations in the participation rate and control of other variables, more research is needed to accurately describe caffeine consumption in medical trainees. Additionally, it would be beneficial to investigate potentially biased perceptions of fatigue and work performance among residents who state caffeine has a limited effect on concentration.

## Conclusions

The data reflects no significant difference between medical and surgical residents or difference in usage and effects of caffeine along years of training. However, there is a clinically significant difference when comparing the usage and effects of caffeine between medical students and all resident populations.

## Appendices

Survey

Sex: male, female, non-binary/third gender, prefer not to say

Age: <20 years old, 20-24 years old, 25-29 years old, 30-35 years old, >35 years old

Education year: MS1, MS2, MS3, MS4, PGY1, PGY2, PGY3, PGY4, ≥PGY5

Program: medical student, surgery/surgical sub-specialty resident, medicine/specialist resident, other

How much caffeine do you consume daily (8-oz cup of coffee = 80-100 mg, 1 Celsius = 200 mg, pre-workout = on average 300 mg)?: none at all, <100mg, 100-199 mg, 200-299 mg, 300-399 mg, ≥400 mg

In a typical week, which of the following sources of caffeine do you use (check one or more)?: coffee, energy drinks, tea, supplement/pills, pre-workout, soda, other, none

How often do you feel tired during the day?: never, rarely, sometimes, often always

How many days per week do you experience difficulties focusing on the task at hand?: never, 1-2 days per week, 3-4 days per week, 4-6 days per week, 7 days per week

On average, how many hours of sleep do you get per night?: <2 hours, 2-4 hours, 4-6 hours, 6-8 hours, 8-10 hours, >10 hours

I consider my nightly sleep quality as: very poor, poor, adequate, good, very good

How has your caffeine intake changed since starting your current level of training?: no change, increased, decreased

When do you consume the bulk of your caffeine?: morning, afternoon, evening, all day long, N/A
